# Inpatient Z-drug use commonly exceeds safe dosing recommendations

**DOI:** 10.1371/journal.pone.0177645

**Published:** 2017-05-16

**Authors:** Todd C. Lee, André Bonnici, Robyn Tamblyn, Emily G. McDonald

**Affiliations:** 1 Department of Medicine, McGill University Health Centre, Montréal, Canada; 2 Clinical Practice Assessment Unit, McGill University Health Centre, Montréal, Canada; 3 Department of Epidemiology, Biostatistics, and Occupational Health, McGill University, Montréal, Canada; 4 Pharmacy Department, McGill University Health Centre, Montréal, Canada; Royal College of Surgeons in Ireland, IRELAND

## Abstract

**Importance:**

In 2016 recommendations for safer prescribing practices were circulated to all doctors in one of Canada’s largest provinces, by the college of physicians, following a coroner’s inquest into a vehicular death related to Z-drug use. We sought to determine how frequently Z-drug prescriptions in our institution were not adhering to these recommendations.

**Design:**

Retrospective cohort study.

**Setting:**

McGill University Health Centre, an 832-bed tertiary care institution in Montréal, Canada.

**Participants:**

All adult non-obstetrical patients admitted between April 1, 2015 and March 31, 2016.

**Exposure:**

The receipt of at least one dose of Z-drug as determined by pharmacy records.

**Main outcomes and measures:**

Adherence to four recommendations related to starting dose, maximal dose, concomitant drug administration, and duration of use were evaluated.

**Results:**

1,409 unique patients received a Z-drug during 1,783 admissions representing use in 9.3% of non-obstetrical patients. Standing orders were seen in 42% (745/1783) of admissions. Non-conformity with the coroner’s recommendations was common. Overall, 672/1783 (38%) admissions involved a patient receiving more than the recommended daily maximum dose (643/999 older patients, 64%). Of 607 admissions which were longer than 10 days, 257 (39%) involved a prescription which exceeded 10 days.

**Conclusions and relevance:**

A coroner’s recommendation that doctors receive instructions about safe Z-drug prescribing is unprecedented, and was likely required given that use of Z-drugs occurs at doses and durations that often exceed best practice recommendations. Similar interventions may be required in other jurisdictions.

## Introduction

Z-drugs (including zopiclone, eszopiclone, zolpidem and zaleplon) belong to a group of non-benzodiazepine sedative-hypnotics that are used in the treatment of insomnia. In the United States, the use of Z-drugs has increased over a 15-year period (1993–2007) with prescriptions increasing 5 times more rapidly than documented diagnoses of insomnia[1]. In Canada, although many drugs are covered by provincial drug plans, in Québec, Z-drugs are not subsidized, and so historically use has been relatively infrequent[2]. However, these medications are still prescribed to patients whose private insurers cover them or to those who are willing to pay out of pocket.

With Z-drug consumption on a dramatic rise, concerns have mounted regarding their safety and misuse[3]. Though perceived to have an improved safety profile over benzodiazepines, Z-drugs are not benign and in observational studies have been linked with dementia, delirium and serious risk of injurious fall[4]. Across North America there have been several safety warnings related to this class of medications, and in particular related to their use in older patients[5]. Believed to have a significant potential for harm, and with very little evidence for benefit on sleep quality, duration or latency[4], the Food and Drug Administration produced recommendations for eszopiclone limiting its dose and duration (Table 1)[6]. In Canada, in May 2016, Québec’s provincial college of physicians posted an info-letter informing all doctors in one of the country’s largest provinces (population 8.2 million) of important precautions for safer prescribing of zopiclone[7]. Finally, an alert was distributed in response to the Québec coroner office’s recommendations, following a vehicular death related to the use of a Z-drug. This unprecedented recommendation mirrored a “Dear Healthcare Professional Letter” sent out by the drug’s manufacturer and by Health Canada in November 2014[8]. The letter contained information related to limiting the starting dose, using the lowest effective dose, not exceeding a maximum dose in older patients or in those with renal or hepatic impairment, or concomitant CYP3A4 inhibitor use, highlighting a need for dose adjustment with concomitant CNS depressing drugs, and that use should generally not exceed 7–10 days[6].

**Table 1:** Conformity to Z-drug prescribing recommendations.

Zopiclone Recommendation	Eszopiclone Recommendation (for comparison)	Proportion Potentially Non-Conforming
The starting dose should be limited to 3.75mg and can be increased as needed to the minimum effective dose not exceeding 7.5mg	The starting dose should be limited to 1mg and can be increased as needed to the minimum effective dose not exceeding 3mg	373/499 (75%) who received a first dose after 72 hours exceeded 3.75mg
The dose should not exceed 5mg in patients who are older or who have renal or hepatic impairment	The dose should not exceed 2mg in older patients and in those with severe liver disease	643/999 (64%) Age 65 or older exceeded 5mg
		210/362 (58%) Age 80 or older exceeded 5 mg
		***From the cohort study (102 Users in 2180 Admissions)*:**
		13 (13%) had stage III or greater renal impairment • 6 (50%) were on dialysis
		8 (8%) had cirrhosis
Dose adjustment may be necessary with concomitant CNS depressing drugs	Not specifically addressed	***From the cohort study (102 Users in 2180 Admission)*:**
		21 (21%) received concomitant narcotics
		20 (20%) received concomitant benzodiazepines
Use should generally not exceed 7–10 days. Use for more than 2–3 consecutive weeks requires complete re-evaluation of the patient.	Not specifically addressed	257/607 (39%) with stays longer than 10 days received 10 or more days of treatment • 171/257 (68%) was standing use
Patients should be instructed to wait for at least 12 hours after dosing before driving or engaging in other activities requiring full mental alertness, especially for older patients and for patients who take the 7.5 mg dose.	Patients taking the 3mg dose are cautioned against driving or engaging in other hazardous activities which need mental alertness the day after use	N/A

There are few guidelines addressing appropriate prescribing for sedative hypnotics in hospitalized patients. However, harms are evident and sedative hypnotic use in hospitalized patients has been independently linked to increased odds of cardiopulmonary arrest[9]. Importantly, when compared to community dwelling adults, hospitalized patients are frequently frail and would be expected to be as vulnerable, if not more-so, to the adverse effects of pharmacologic treatment of insomnia.

We hypothesized that the availability of zopiclone on our hospital’s formulary was leading to a high prevalence of in-patient exposure, propagation of outpatient prescriptions, and potentially unsafe prescribing practices in a vulnerable, older population. In light of the coroner’s recommendations, we sought to determine how frequently zopiclone is prescribed in our institution outside of available safety recommendations.

## Methods

This retrospective cohort study took place at the McGill University Health Centre, an 832-bed tertiary care institution (catchment area 850,000) in Montréal, Canada. From the pharmacy database, we obtained anonymized inpatient prescription data for all patients who received at least one dose of zopiclone (the only Z-drug on formulary) during fiscal year 2015 (April 1, 2015 to March 31, 2016). Obstetrical admissions were excluded. Z-drugs are not included on any of our hospital’s pre-printed admission orders.

We categorized patients as medical or non-medical based on the unit they were admitted to. Use was categorized as PRN or standing, based on the initial order. Determination of the total potentially deliverable daily dose assumed that PRN doses were given. The first prescribed dose and the maximum prescribed dose were also recorded.

Each recommendation (other than the subjective use of the lowest effective dose) was examined separately and in the following manner (summarized in Table 1).

### Recommendation 1: The recommended daily starting dose should not be exceeded

We defined *new use* as patients who received the drug for the first time following 72 hours of hospitalization. We did not have access to pre-hospitalization outpatient prescriptions.

### Recommendation 2: The maximum dose should be limited in patients who are older who have renal or hepatic impairment, or who are taking CYP3A4 inhibitors

We defined older adults as age 65 or above and estimated the proportion of medical patients with concomitant hepatic or renal disease who received zopiclone by manually extracting comorbidities from the electronic health record when available[10]. Concomitant use of CYP3A4 inhibitors was not available.

### Recommendation 3: Dose adjustment may be necessary with concomitant Central Nervous System (CNS) depressing drugs

We estimated the proportion of medical in-patients who received concomitant CNS depressants (narcotics, including morphine, dilaudid, oxycodone, fentanyl, and codeine and/or benzodiazepines, including lorazepam, clonazepam, oxazepam, and diazepam).

### Recommendation 4: Use should generally not exceed 7–10 days

We limited this evaluation to patients whose length of stay exceeded 10 days and determined the proportion who were prescribed more than 10 consecutive days of the drug.

For patients where post-hospitalization discharge prescription data was available we determined the proportion that were receiving a Z-drug on admission, in the hospital, and at discharge[11].

The McGill University Health Centre Research Ethics Board approved this study.

## Results

Of 15,150 non-obstetrical patients admitted during the 2015–2016 fiscal year there were 1,409 unique patients who received zopiclone comprising 1,783 admissions (S1 Table). This represents use in 9.3% of patients. The median length of stay was six days (interquartile range 2–16). Standing bedtime orders were seen in 42% (745/1783) of all admissions. Non-conformity with Health Canada and manufacturer recommendations was common (Table 1).

Recommendation 1: the recommended daily starting dose should not be exceeded. Of 499 patients who first received zopiclone following 72 hours of admission, 373 (75%) received a prescription that was higher than the recommended starting dose.

Recommendation 2: the maximum dose should be limited in patients who are older who have renal or hepatic impairment. Overall 797/1409 (57%) of patients prescribed zopiclone were over the age of 65; with nearly one-fifth (n = 277) of use occurring in patients older than 80. Overall, 672/1783 (38%) of admissions involved a patient receiving more than the recommended daily maximum. This was more prevalent in older patients (643/999, 64%). Amongst those younger than sixty-five, only 3.7% (29/784) received more than the maximum recommended dose.

In an analysis limited to medical admissions, zopiclone was used in 102 of 2180 admissions (4.8%) with 13% having significant concomitant renal impairment and 8% cirrhosis.

Recommendation 3: dose adjustment may be necessary with concomitant CNS depressing drugs. One in five zopiclone users admitted to a medical unit also received concomitant narcotics or benzodiazepines with 7% receiving all three classes of drug.

Recommendation 4: Use should generally not exceed 7–10 days. Of 607 admissions which were longer than 10 days, 249 (39%) received more than 10 consecutive days of zopiclone and 68% (171/249) had standing orders.

Finally, amongst patients admitted to a medical unit, out of 97 patients receiving zopiclone, 84 survived (87% of users, 4.2% of all discharges) and went on to receive a discharge prescription for zopiclone. Based on available exit prescription data (available for 2434 patients) zopiclone was prescribed at discharge on 5.1% of all exit prescriptions. For comparison, a benzodiazepine was present on 17% of exit prescriptions.

## Discussion

We evaluated inpatient Z-drug prescribing practices in the fiscal year following updated 2014 Health Canada recommendations for safer prescribing and considering a recent coroner mandated Québec-wide advisory. We found that zopiclone was used in over 1,700 admissions and that non-conformity to safe prescribing recommendations was very common, particularly with regards to the starting and maximal doses for older adults. Many prescriptions were given as a standing order as opposed to an as needed basis, were excessive in duration, and were co-prescribed in the presence of concomitant narcotics and/or benzodiazepines. Additionally, even though zopiclone is not listed on the provincial formulary, approximately 1 in 20 admissions received an exit prescription.

Nearly 10% of our non-obstetrical hospital population had an inpatient prescription for zopiclone which was propagated in half of them (5%) at discharge. The use of this particular class of sedative-hypnotics has been increasing over time; by comparison, there were fewer than 2000 prescriptions written for zopiclone between 2002 and 2012 at our hospital (data not shown). This increase in use parallels other jurisdictions[12, 13] though ours is the only study involving hospitalized patients and that has included an evaluation of the doses used, the duration of use, and the frequency of specific comorbidities and co-administrations. For example, in Manitoba between 1996 and 2012 outpatient use of Z-drugs increased from 10.9 to 37.0 per 1000 persons and from 7.3 to 20.3 users per 1,000 in those older than 65. Our rate of 51.0 per 1,000 persons discharged may represent a worrisome temporal trend towards the increasing use of Z-drugs over time. Alternatively, the patient demographics of those requiring hospitalization may differ from the average community population, and an established culture of prescribing sleep aids in hospital may also have put our hospitalized patients at higher risk of exposure.

It has previously been shown that exit prescriptions for similar medications, such as benzodiazepines[14, 15] or antipsychotics[14, 16], can precipitate chronic use. This can occur even if these drugs were originally intended for limited duration use due to inadvertent continuation by community prescribers or through the development of patient dependence. Hospitalized older patients are particularly vulnerable to the side-effects of sedative hypnotic use with a number needed to harm of six for motor vehicle accidents, falls, cognitive impairment, fracture and death and limited evidence of meaningful drug efficacy in terms of sleep[17]. This makes our findings particularly concerning for jurisdictions with more liberal outpatient access to Z-drugs; it is likely that use is even more common outside of Québec where access is not limited to those who pay out of pocket or through private insurance. To address these issues our Centre will pilot automatic dose substitutions based on age, as well as automatic stops for new in hospital starts exceeding 10 days.

Our study has several important limitations. We describe a single center experience over a period of one year prior to recommendations from the coroner and based on out-patient dosing recommendations. We were not able to evaluate in-patient recommendations as these do not exist. We focused our evaluation of many of the recommendations on patients admitted to medical units. This patient population is generally older, with more co-morbid disease, and would be expected to be at highest risk of adverse drug events from Z-drugs. At worst, we have underestimated the magnitude of the problem, as we might expect in-patient recommendations to be even more restrictive than those in existence for community-dwellers. Our results may not be generalizable to other centers and future practice within the province may be influenced by the coroner’s recommendations. We only had access to a limited number of comorbidities, co-administrations, and exit prescriptions for a subset of medical in-patients and this data did not completely cover the same time period as pharmacy data. As such, our estimates in these areas may not be representative of our population as a whole. In addition, we did not have access to concomitant use of CYP3A4 inhibitors. Finally, while we know that patients left the hospital with an exit prescription for zopiclone we do not know if they filled it or for how long it was continued post-discharge.

## Conclusions

Despite many advances towards improved prescribing practices in older patients, the use of sedative-hypnotics such as Z-drugs is common, even in frail, vulnerable, hospitalized patients. We describe doses and durations that often exceeded available best practice recommendations. Use occurred in patients receiving other CNS depressants and in those with relative co-morbid contraindications, increasing the risk of adverse drug events. Finally, use was continued at discharge in more than half of those exposed. Given the limited utility of these potentially harmful medications in older patients, we call on other hospitals to evaluate their prescribing practices and for well-designed studies to better inform prescribers of the benefits and harms of Z-drugs or similar medications.

## Supporting information

S1 Table (XLS)
